# Supplementary material for: Casein Kinase 1D Encodes a Novel Drug Target in Hedgehog—GLI-Driven Cancers and Tumor-Initiating Cells Resistant to SMO Inhibition
Source: Cancers (Basel). 2021 Aug 23;13(16):4227. doi: 10.3390/cancers13164227 (PMC8394935; doi:10.3390/cancers13164227)
Supplement: Supplementary file 1 [file cancers-13-04227-s001.zip › cancers-1284320-supplementary.pdf]

cancers

Supplementary Material: Casein Kinase 1D Encodes a Novel Drug Target in Hedgehog–GLI-Driven Cancers and Tumor-Initiating Cells Resistant to SMO Inhibition

Elisabeth Peer, Sophie Karoline Aichberger, Filip Vilotic, Wolfgang Gruber, Thomas Parigger, Sandra Grund-Gröschke, Dominik Patrick Elmer, Florian Rathje, Andrea Ramspacher, Mirko Zaja, Susanne Michel, Svetlana Hamm and Fritz Aberger

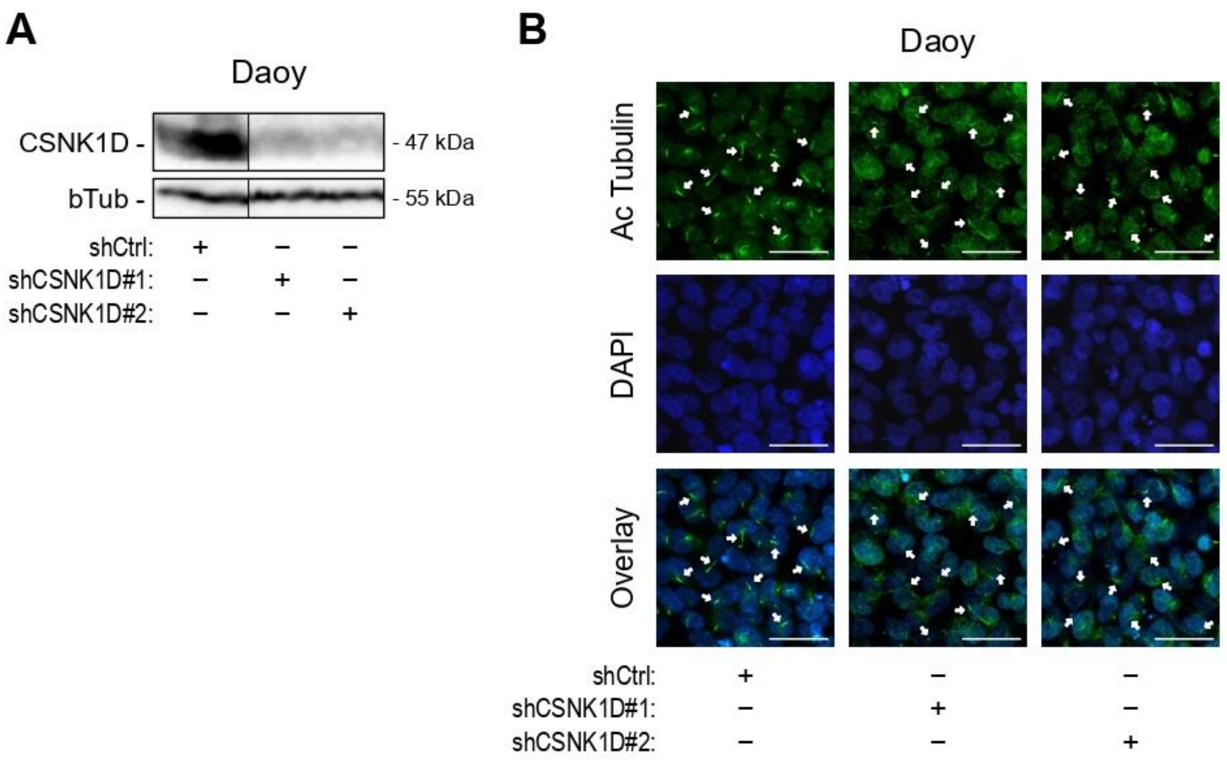

Figure S1. Targeting CSNK1D does not interfere with ciliogenesis in Daoy cells. **(A)** Representative. Western blot analysis of CSNK1D in Daoy cells lentivirally transduced with shCSNK1D (#1, #2) or control shRNA (shCtrl). **(B)** Confocal imaging of primary cilia in Daoy cells lentivirally transduced with shCSNK1D (#1, #2) or control shRNA (shCtrl). For visualization of primary cilia (indicated by white arrows) Daoy cells were stained with antibodies against acetylated tubulin as described previously [1]. Scale bars: 50 µm.

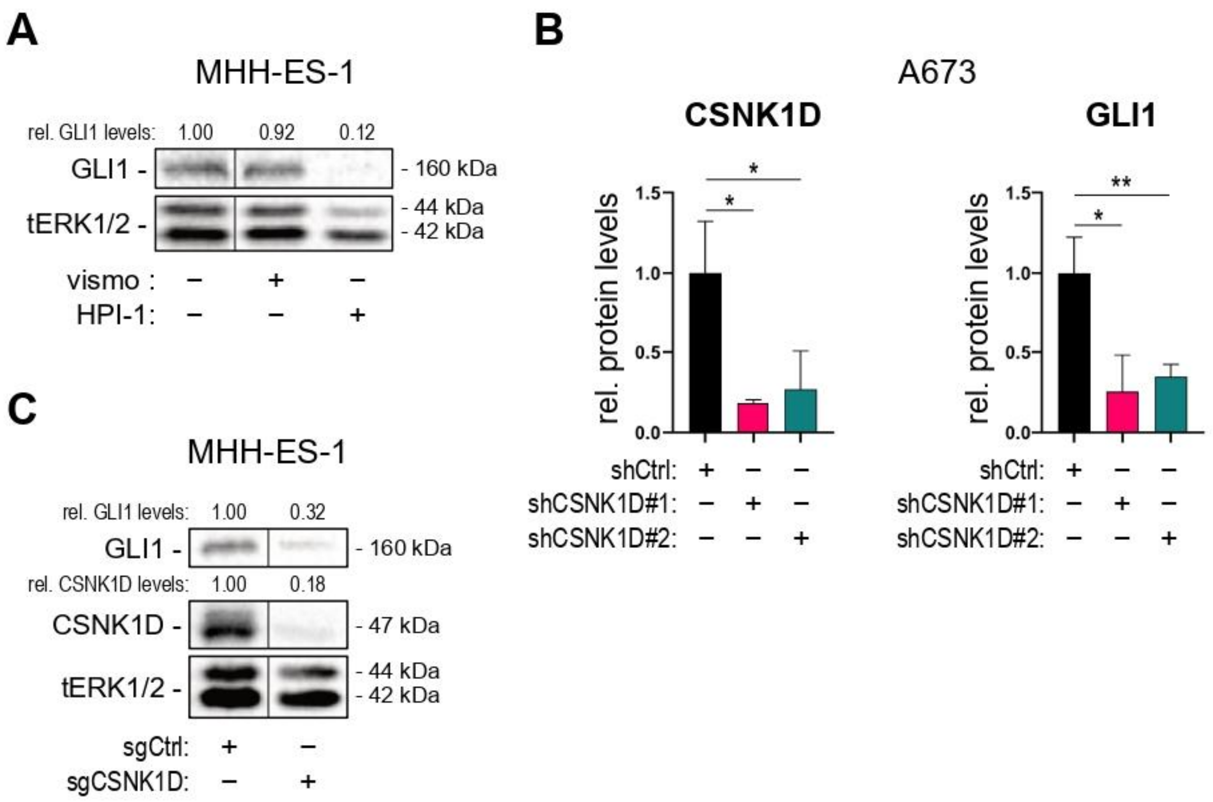

Figure S2. Targeting CSNK1D interferes with oncogenic HH/GLI signaling in SMOi resistant Ewing. Sarcoma cells. **(A)** Representative Western blot analysis of GLI1 in MHH-ES-1 cells treated with vismodegib [1 μM] or HPI-1 [20 μM]. **(B)** Quantification of relative GLI1 protein levels in A673 cells lentivirally transduced with shCSNK1D (#1, #2) or control shRNA (shCtrl) (n = 3). **(C)** Representative Western blot analysis of GLI1 in MHH-ES-1 cells lentivirally transduced with sgCSNK1D or sgCtrl. Quantification of Western blot bands was conducted via densitometric image analysis using Image Lab 5.0 software (Bio-Rad). Relative protein levels normalized to the loading control total ERK (tERK) and to the Ctrl condition are indicated above each protein band. Student's t test was used for statistical analysis (*P < 0.05; **P < 0.01).

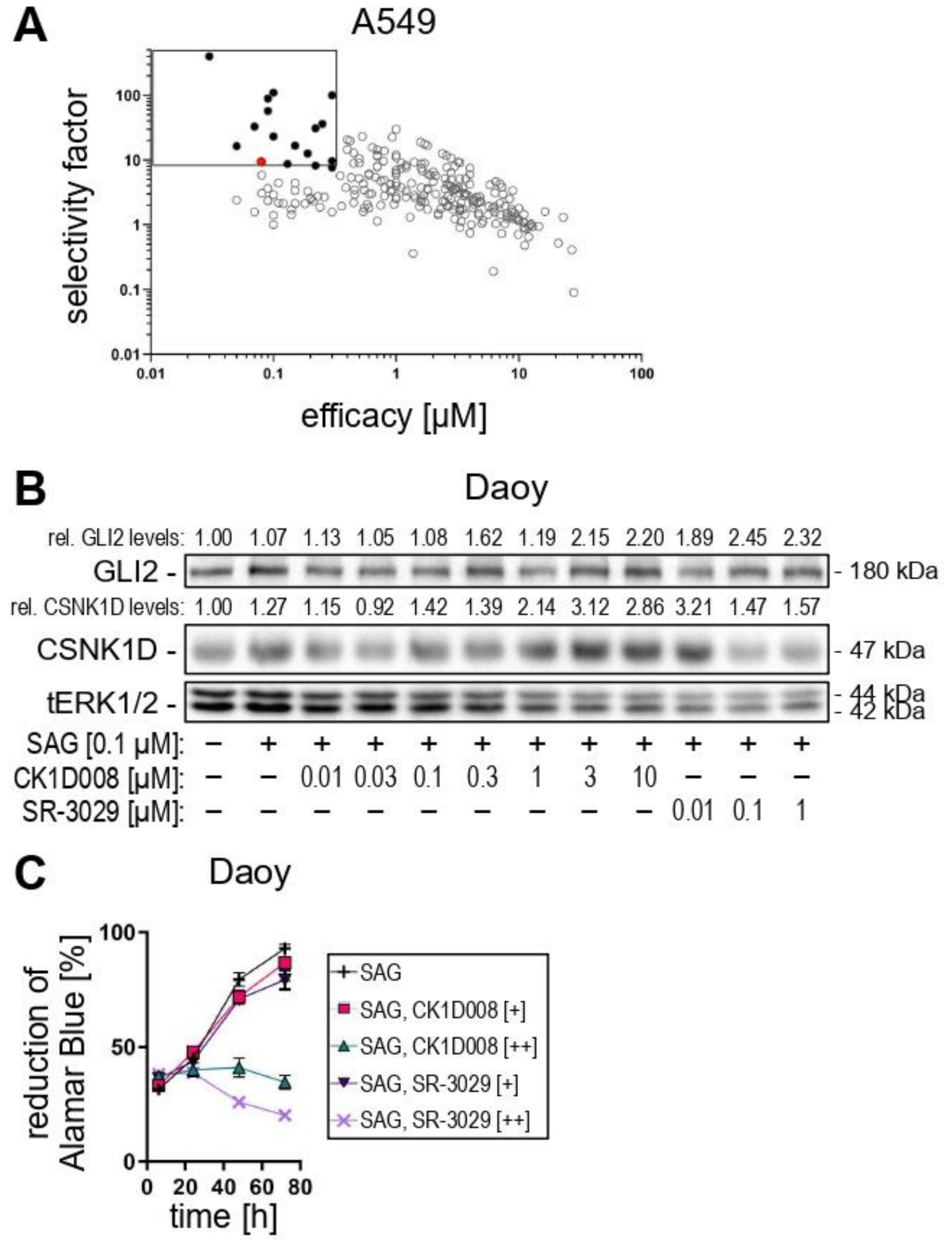

Figure S3. Effects of pharmacological targeting of CSNK1D on the proliferation of A549 and Daoy cells. **(A)** Cellular compound screening cascade in A549 cells. Compounds (black dots) with an IC50 below 0.3 µM under non-proliferating conditions (= efficacy) and a selectivity factor of at least 10 were selected for further evaluation. CK1D008 is highlighted by a red dot. **(B)** Representative Western blot analysis of GLI2 and CSNK1D protein levels in Daoy cells treated with SAG [100 nM] and increasing concentrations of CK1D008 [0.01 – 10 µM] or SR-3029 [0.01 – 1 µM]. **(C)** Daoy cells were treated with SAG [100 nM] and increasing concentrations of CK1D008 ([0.3 µM] (+), [3 µM] (++)] or SR-3029 ([0.1 µM] (+), [1 µM] (++)) and proliferation was assessed in AlamarBlue assays.

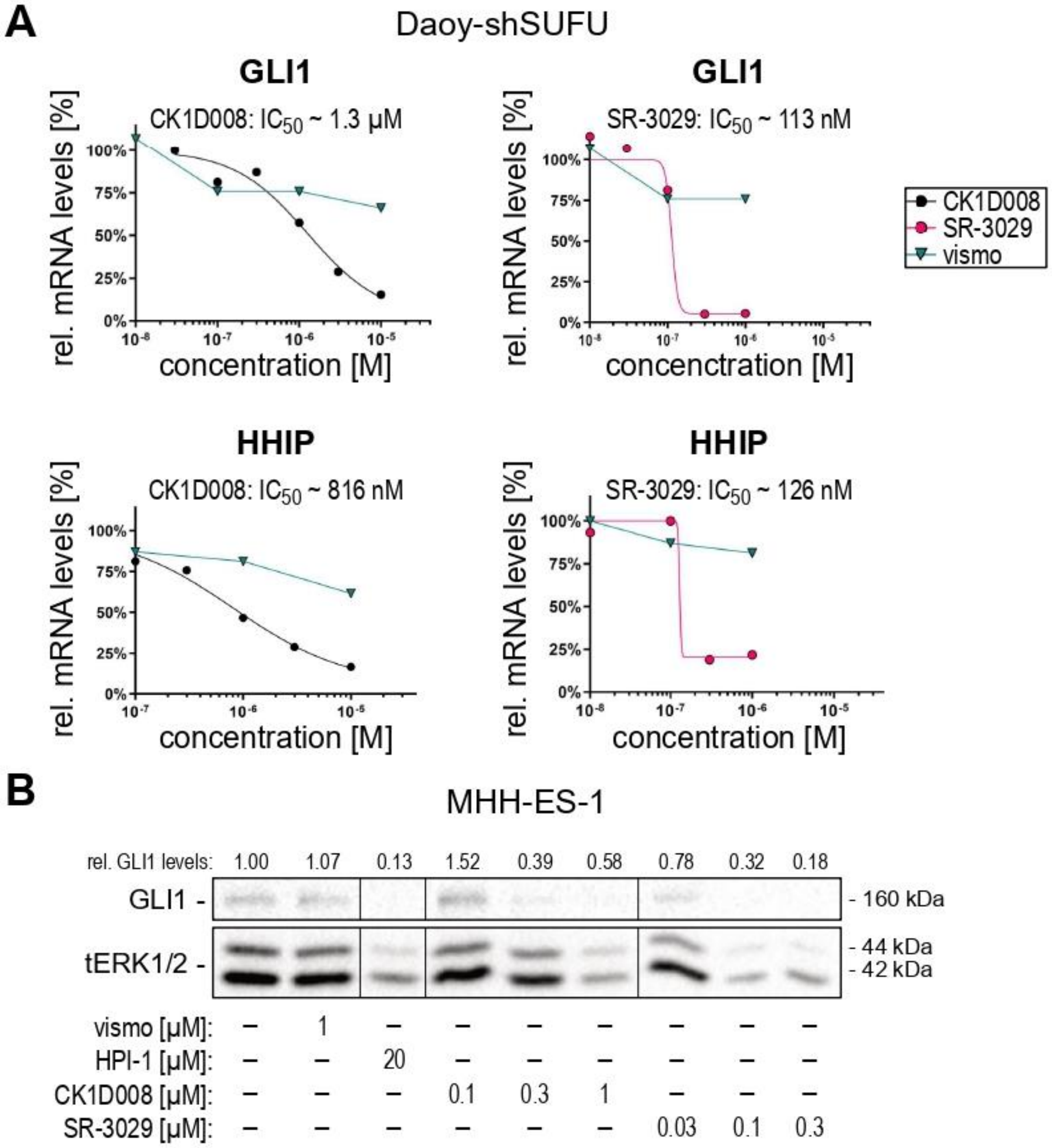

Figure S4. Targeting of CSNK1D reduces HH target gene expression in SMOi resistant cell lines. **(A)** CK1D008 and SR-3029 reduce HH/GLI target gene expression in Daoy-shSUFU medulloblastoma cells with IC50 values in the low micromolar or nanomolar range, respectively. mRNA expression levels of GLI1 and HHIP were analyzed by qPCR and expressed as percentage relative to the control condition. **(B)** Representative Western blot analysis of GLI1 in MHH-ES-1 cells treated with vismo [1 µM], HPI-1 [20 µM], CK1D008 [0.1 – 1 µM] or SR-3029 [0.03 – 0.3 µM]. Relative quantification of Western blot bands was conducted via densitometric image analysis using Image Lab 5.0 software (Bio-Rad). Relative protein levels normalized to the loading control total. ERK (tERK) and to the Ctrl condition are indicated above each protein band.

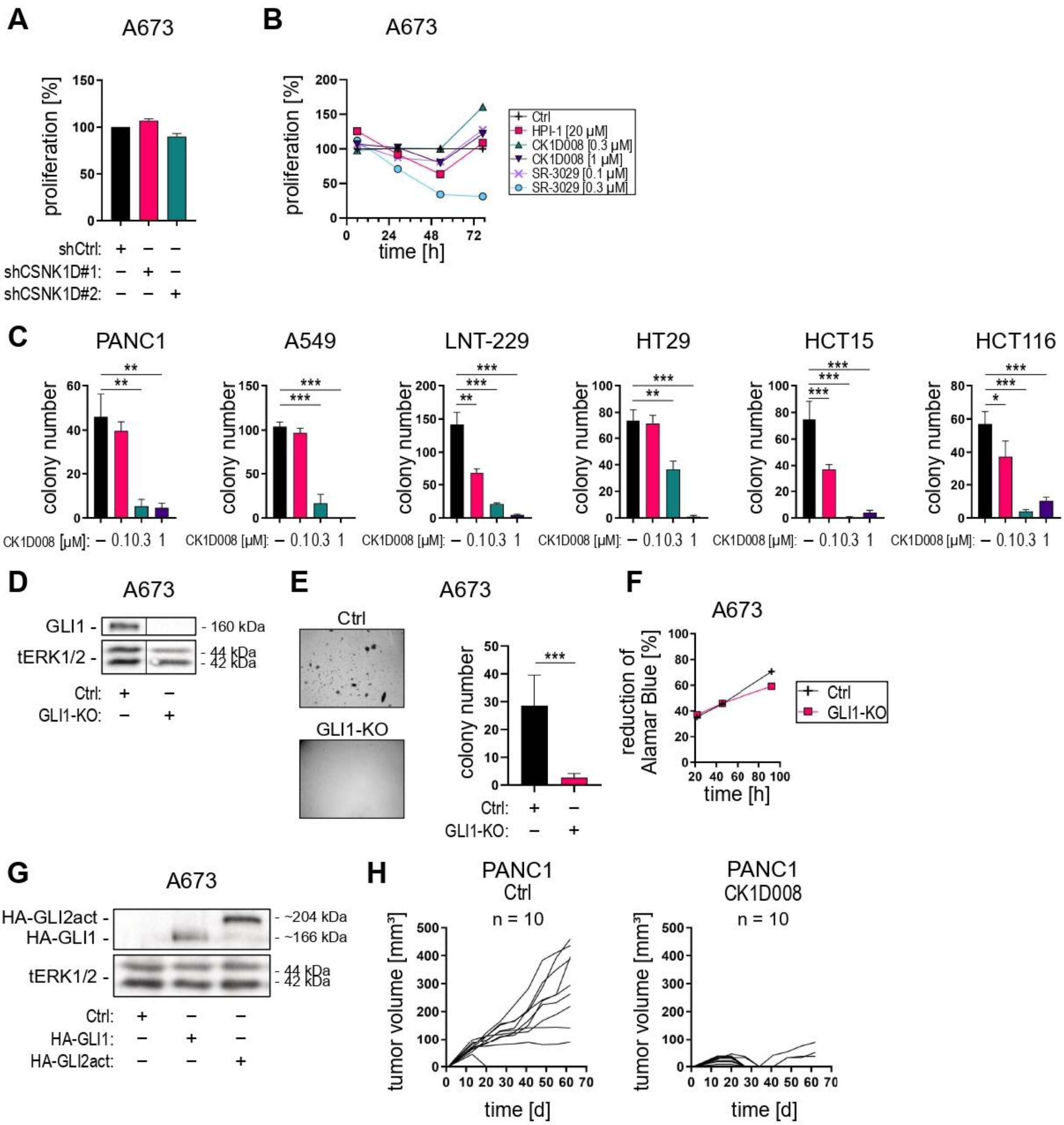

Figure S5. Pharmacologic and genetic inhibition of CSNK1D selectively abrogates clonal growth and impairs *in vivo* engraftment of GLI-dependent PANC-1 cancer cells. **(A)** A673 cells were lentivirally transduced with shCSNK1D (#1, #2) or control shRNA (shCtrl) and proliferation was assessed in an AlamarBlue assay (n = 3). **(B)** A673 cells were treated with HPI-1 [20 µM], CK1D008 [0.3 µM, 1 µM] or SR-3029 [0.1 µM, 0.3 µM] and proliferation was assessed in an AlamarBlue assay. **(C)** PANC1, A549, LNT-229, HT29, HCT15 and HCT116 cells were pre-treated with CK1D008 [0.1 µM, 0.3 µM, 1 µM] for 48h, seeded in limited dilutions and cultured under anchorage-dependent non-proliferating conditions. Colony number was assessed after 9-11 days (n = 3). **(D)** Representative Western blot analysis of GLI1 protein levels in A673 cells lentivirally transduced with sgGLI1 or sgCtrl. **(E)** Knockout of GLI1 reduces anchorage-independent growth of A673 cells in soft agar. Representative images of formed anchorage-independent 3D tumorspheres (left panel), number of colonies (right panel) (n = 5). **(F)** A673 cells were lentivirally transduced with sgGLI1 or sgCtrl and proliferation was assessed in an AlamarBlue assay. **(G)** Representative Western blot analysis of overexpressed HA-tagged GLI1 and HA-tagged GLI2act protein levels in A673 cells. **(H)** PANC1 cells were pre-treated with CK1D008 [0.3 µM] for 48h. 1x106 viable Ctrl or CK1D008 pre-treated PANC1 cells were engrafted in nude mice and tumor volume was monitored (n = 10). Student's t test was used for statistical analysis (*P < 0.05; **P < 0.01; ***P < 0.001).

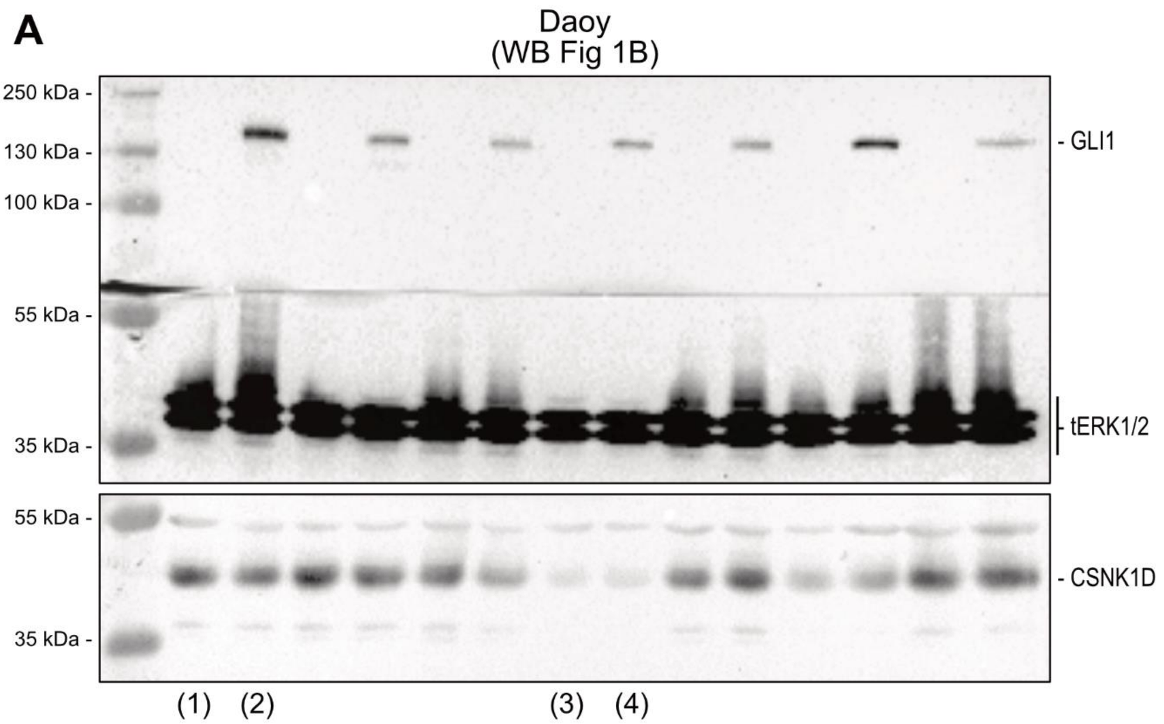
A
Daoy
(WB Fig 1B)
250 kDa -
130 kDa -
100 kDa -
55 kDa -
35 kDa -
- GLI1
tERK1/2
55 kDa -
35 kDa -
- CSNK1D
(1) (2) (3) (4)

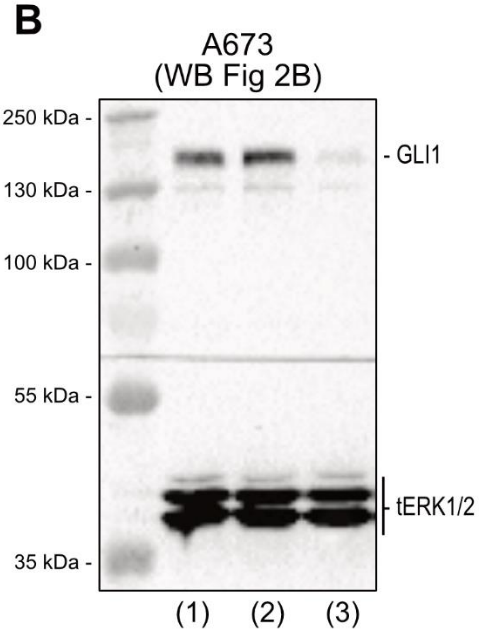
B
A673
(WB Fig 2B)
250 kDa -
130 kDa -
100 kDa -
55 kDa -
35 kDa -
- GLI1
tERK1/2
(1) (2) (3)

C

A673
(WB Fig 2C left)

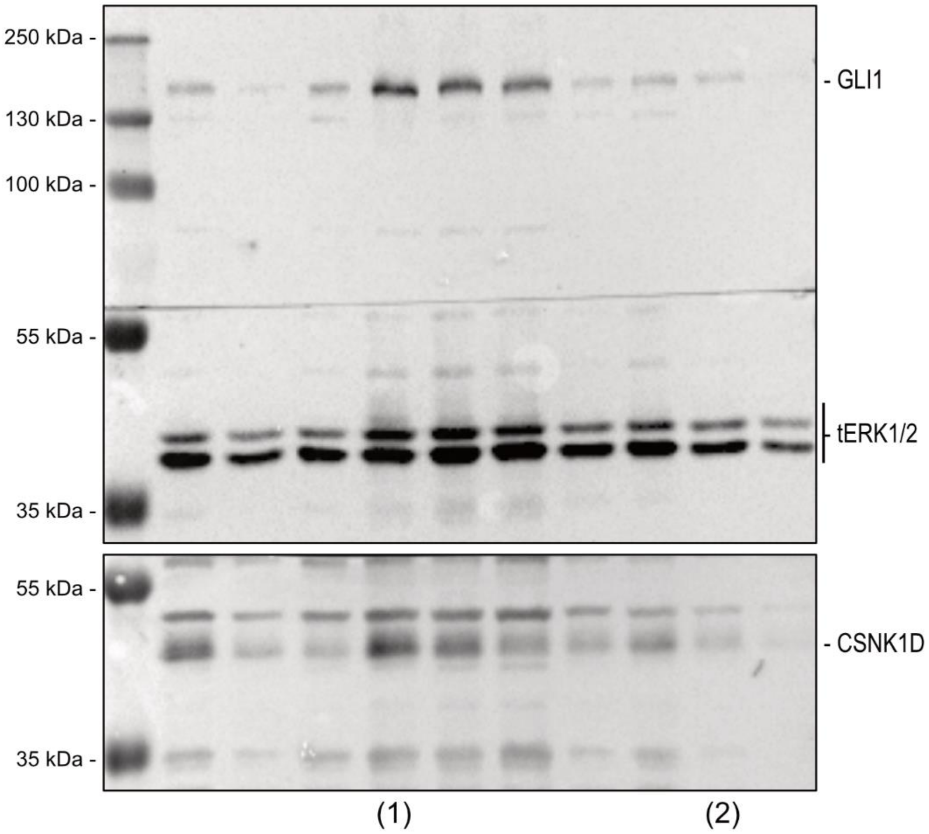

A673
(WB Fig 2C right)

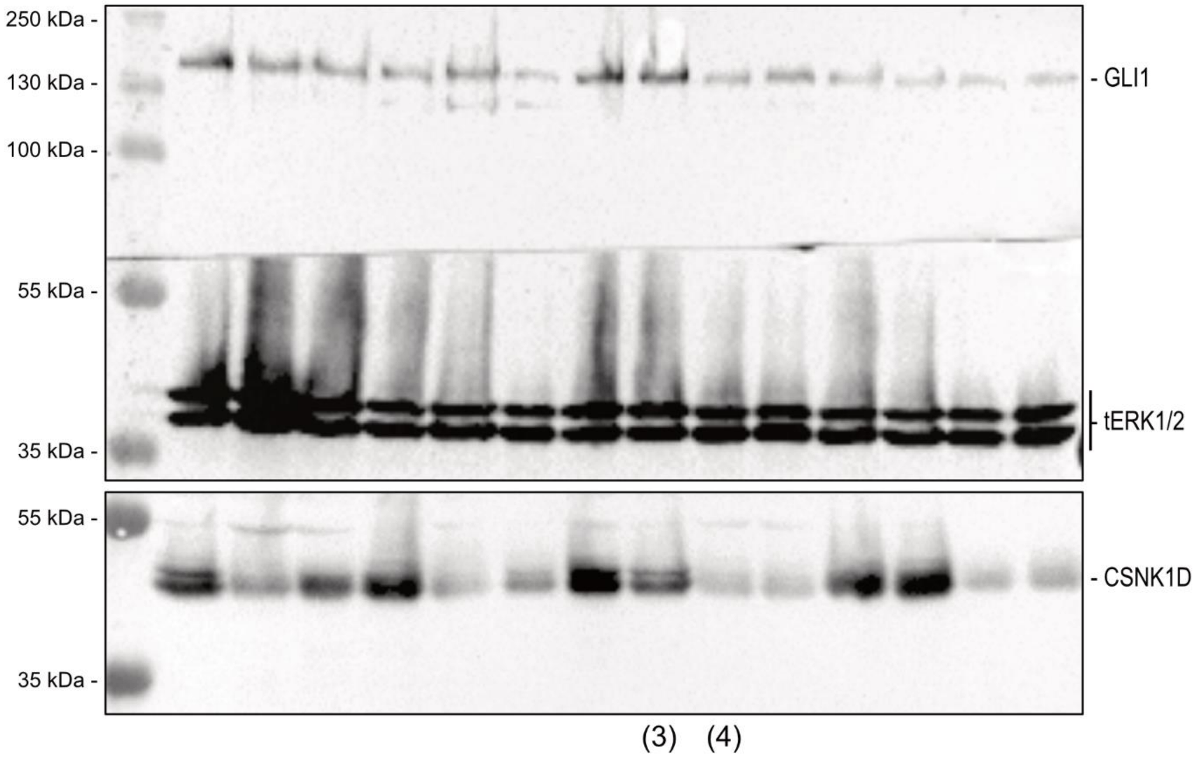

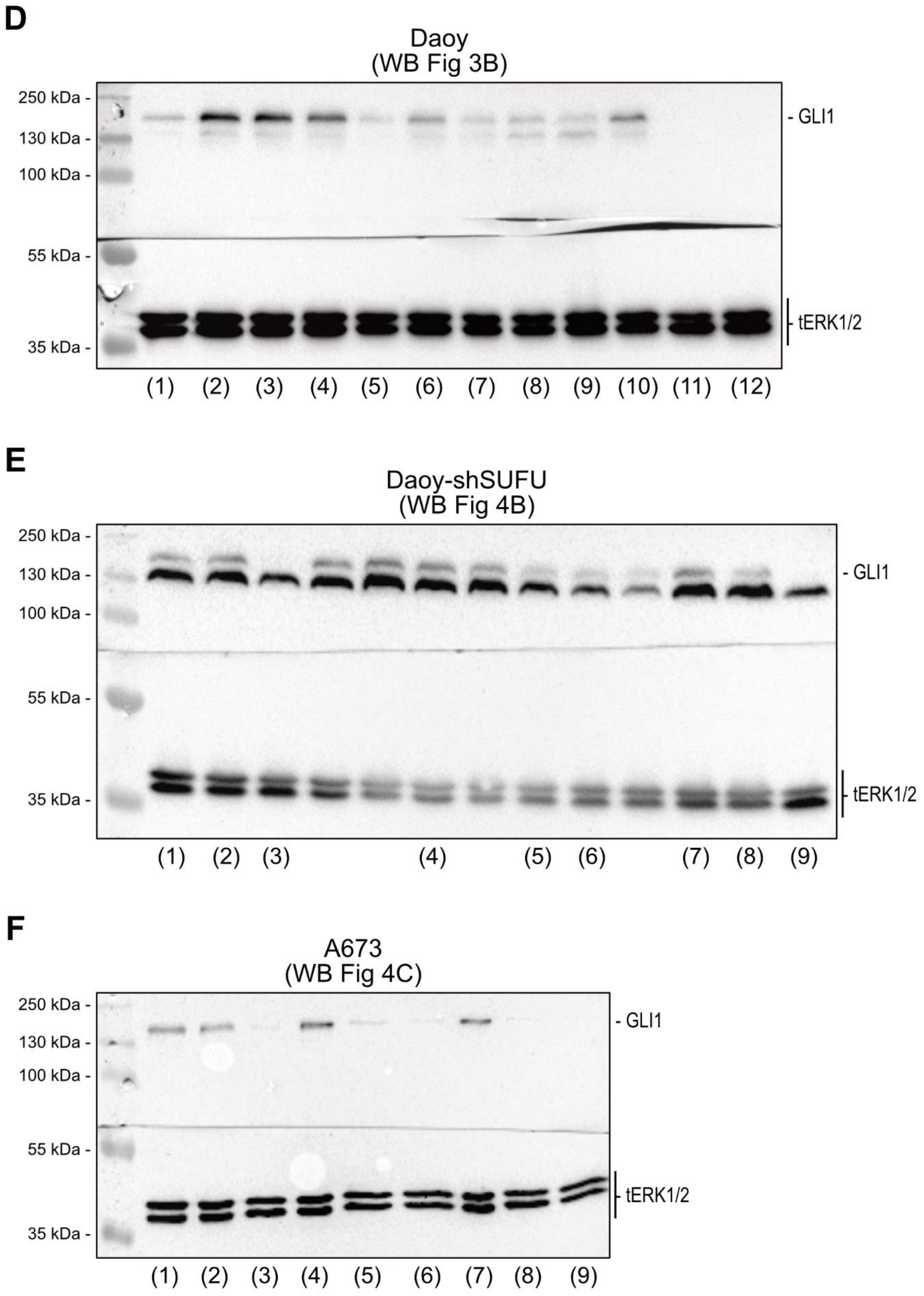

Figure S6. Original Western Blot.

Table S1. qPCR cycling program.

Temperature	Duration	Type
95 °C	3 min	Hold
95 °C	10 sec	Cycling 40x
65 °C	15 sec	
72 °C	30 sec	
Melting Curve		
Ramp from 70 °C to 95 °C rising by 0.5 °C each step	Wait for 45 sec on first step, Wait for 5 sec for each step afterwards	Melting curve analysis

Table S2. Western blot antibodies.

Target (clone)	Supplier	Dilution
anti-GLI1 (V812)	Cell Signaling, Danvers, MA, United States	1:1000
anti-p44/42 MAPK (Erk1/2; tERK)	Cell Signaling, Danvers, MA, United States	1:1000
anti-HA-Tag (C29F4)	Cell Signaling, Danvers, MA, United States	1:1000
anti-β-Tubulin (9F3)	Cell Signaling, Danvers, MA, United States	1:1000
anti-CSNK1D (AF12G4)	abcam, Cambridge, United Kingdom	1:5000
anti-GLI2 (H-300)	Santa Cruz, Dellas, TX, United States	1:1000
anti-rabbit IgG, HRP-linked	Cell Signaling, Danvers, MA, United States	1:3000
anti-mouse IgG, HRP-linked	Cell Signaling, Danvers, MA, United States	1:3000

References:

1. Gruber, W.; Hutzinger, M.; Elmer, D.P.; Parigger, T.; Sternberg, C.; Cegielkowski, L.; Zaja, M.; Leban, J.; Michel, S.; Hamm, S.; et al. DYRK1B as therapeutic target in Hedgehog/GLI-dependent cancer cells with Smoothened inhibitor resistance. *Oncotarget* **2016**, *7*, 7134-7148, doi:10.18632/oncotarget.6910
